# A Key Role for Nectin-1 in the Ventral Hippocampus in Contextual Fear Memory

**DOI:** 10.1371/journal.pone.0056897

**Published:** 2013-02-13

**Authors:** Martina Fantin, Michael A. van der Kooij, Jocelyn Grosse, Claude Krummenacher, Carmen Sandi

**Affiliations:** 1 Laboratory of Behavioral Genetics, Brain Mind Institute, School of Life Sciences, Ecole Polytechnique Fédérale de Lausanne EPFL, Lausanne, Switzerland; 2 Department of Pathobiology, University of Pennsylvania School of Veterinary Medicine, Philadelphia, Pennsylvania, United States of America; Université Pierre et Marie Curie, France

## Abstract

Nectins are cell adhesion molecules that are widely expressed in the brain. Nectin expression shows a dynamic spatiotemporal regulation, playing a role in neural migratory processes during development. Nectin-1 and nectin-3 and their heterophilic trans-interactions are important for the proper formation of synapses. In the hippocampus, nectin-1 and nectin-3 localize at puncta adherentia junctions and may play a role in synaptic plasticity, a mechanism essential for memory and learning. We evaluated the potential involvement of nectin-1 and nectin-3 in memory consolidation using an emotional learning paradigm. Rats trained for contextual fear conditioning showed transient nectin-1—but not nectin-3—protein upregulation in synapse-enriched hippocampal fractions at about 2 h posttraining. The upregulation of nectin-1 was found exclusively in the ventral hippocampus and was apparent in the synaptoneurosomal fraction. This upregulation was induced by contextual fear conditioning but not by exposure to context or shock alone. When an antibody against nectin-1, R165, was infused in the ventral-hippocampus immediately after training, contextual fear memory was impaired. However, treatment with the antibody in the dorsal hippocampus had no effect in contextual fear memory formation. Similarly, treatment with the antibody in the ventral hippocampus did not interfere with acoustic memory formation. Further control experiments indicated that the effects of ventral hippocampal infusion of the nectin-1 antibody in contextual fear memory cannot be ascribed to memory non-specific effects such as changes in anxiety-like behavior or locomotor behavior. Therefore, we conclude that nectin-1 recruitment to the perisynaptic environment in the ventral hippocampus plays an important role in the formation of contextual fear memories. Our results suggest that these mechanisms could be involved in the connection of emotional and contextual information processed in the amygdala and dorsal hippocampus, respectively, thus opening new venues for the development of treatments to psychopathological alterations linked to impaired contextualization of emotions.

## Introduction

Nectins are immunoglobulin-like adhesion molecules that connect cells. Four different nectin types, nectin 1–4, have been described so far [1]. In the central nervous system, these cell adhesion molecules aggregate in formations, termed puncta adherentia junctions, which are mechanical adhesive sites that connect pre- and postsynaptic membranes [2].

In the hippocampus, nectin-1 has been found to be preferentially localized in axons, while its main heterophilic partner, nectin-3, has been detected in axons and dendrites in both neuronal cultures [3] and *in vivo* [4]. Nectin-1 and nectin-3 knockout (KO) mice have a reduced number of puncta adherentia junctions and display abnormalities in the mossy fiber trajectories of the CA3 region of the hippocampus [5]. Cultured neurons from nectin-1 KO mice showed altered dendritic spine morphology [3]. Recently, it was shown that nectin-1 regulates spine density in hippocampal neurons through ectodomain shedding [6]. The spatiotemporal expression of Nectin-1 is dynamic; during the neonatal period this cell-cell adhesion molecule is localized in brain regions associated with inter-hemispheric connections (corpus callosum, hippocampus, anterior commissure and associated cortical structures) while during adulthood its expression is more restricted to limbic-related structures [7]. In the neonatal brain, nectin-1 expressing cells found in the corpus callosum and developing cerebral cortex display a typical migratory phenotype and nectin-1 is therefore thought to be involved in migratory processes during neurodevelopment [7], [8].

Changes in hippocampal synaptic plasticity morphology have been implicated in a number of learning paradigms, including spatial navigation [9], [10], passive avoidance [11] and contextual fear conditioning (CFC) [12], [13], [14]. CFC takes place when a neutral context is associated with an aversive unconditioned stimulus. The unconditioned stimulus (e.g., a footshock) by itself elicits a fear response, comprising both autonomic and behavioral (e.g., freezing) responses, and after conditioning, the context becomes an aversive stimulus that predicts threat and induces a conditioned state of fear and associated responses. CFC induces a robust form of emotional memory that is dependent on intact hippocampal and amygdala function. Importantly, while the amygdala is also involved in unimodal conditioning, such as auditory cue-shock associations, the role of the hippocampus has been associated with multimodal context-shock associations [15], [16], [17]. However, the hippocampus is not a unitary structure. Increasingly, a functional, morphological and molecular segmentation along the dorsal-ventral axis is being recognized [18], [19]. Although some studies have pointed out the specific involvement of the dorsal [20], [21], [22], but not ventral, hippocampus in contextual fear memory formation [20], [23], accumulated evidence indicates a role for the ventral hippocampus in the formation [24], [25], [26], [27], [28] and expression [20], [29], [30], [31] of contextual fear memory. The emerging view is that the respective roles of the ventral and dorsal hippocampus in contextual fear conditioning might differ, with the ventral part being particularly involved in the processing of fear and anxiety processes, while the dorsal hippocampus is involved in the temporal and contextual aspects of event representation [28]. However, the molecular mechanisms implicated in CFC at each hippocampal compartment are not yet known.

Cell adhesion molecules of the immunoglobulin superfamily have been implicated in memory formation [32], [33], [34] and stress and memory interactions [35], [36], [37], with most of the work focusing on NCAM, its polysialylated form (PSA-NCAM), and L1CAM. In fear conditioning, NCAM KO mice show deficits in contextual [38], [39] and auditory [38], [40] fear memories. Conversely, increased hippocampal expression of NCAM, PSA-NCAM and L1NCAM was observed 24 h after CFC training in rats [41], [42], [43]. CFC memory consolidation was found to be impaired after the infusion of a synthetic peptide that interferes with NCAM function [44], but it was found to be improved after the infusion of another peptide that corresponds to the binding site of NCAM for the fibroblast growth factor receptor 1 [45]. Although no information exists for NCAM or L1CAM regarding their role in different hippocampal areas, modulation of PSA-NCAM expression by CFC was found to be upregulated in the dorsal, but not ventral, hippocampus at the 24 h post-training time point, while PSA depletion in the dorsal, but not ventral, hippocampus resulted in impaired CFC memory consolidation [43].

Although the functional roles of nectins are largely unknown, recent evidence suggests their potential involvement in hippocampal function [46]. Given their role in hippocampal connectivity (see above), we hypothesized a role for nectins in the hippocampus in memory formation for CFC. To test this hypothesis, we evaluated time-dependent changes in the total and synaptoneurosomal expression of nectin-1 and nectin-3 in both the dorsal and ventral hippocampus at different time points following CFC. Identified changes were followed by experiments addressed at dissecting the nature of the changes from a behavioral point of view (i.e., whether the context-shock association was required or the separate components would suffice) and by pharmacological interference with the function of the identified nectin to evaluate the impact of such manipulation in CFC. Additional control experiments were addressed to evaluate potential non-specific memory effects induced by the pharmacological manipulation.

## Materials and Methods

### Animals

Male Sprague-Dawley rats (Charles River Laboratories; Lyon, France)], weighing 250 g at the start of the experiments, were pair-housed under light- (12 h light/dark cycle; lights on at 7:00 A.M.) and temperature (22±2°C)-controlled conditions. Food and water were freely available. All experiments were conducted between 8:30 A.M. and 2:00 P.M. to minimize the influence of hormonal fluctuations. All animals were handled for 2 min/d for the 3 d preceding the first behavioral test or surgery and the two animals from the same home-cage were tested simultaneously. Animal procedures described were conducted in accordance with the guidelines set by the European Community Council Directives (86/609/EEC) and the Swiss Cantonal Veterinary Authorities (Vaud, Switzerland), and approved by the Cantonal Veterinary Office Committee for Animal Experimentation.

### Fear conditioning

Training and testing took place in a rodent observation cage (30×37×25 cm) that was placed in a sound-attenuated chamber. The observation cage was made of stainless steel walls and a Plexiglas door. The floor consisted of a wired mesh that was connected to a shock generator (Panlab, Barcelona, Spain). Each observation cage was cleaned with 0.1% acetic acid before and after each session. Ventilation fans provided background noise of 68 dB, and a 20 W white light bulb illuminated the chamber. On the conditioning/training and testing days, each rat was transported from the colony room to an adjacent experimental room and placed in an observation cage (i.e., the conditioning chamber). The animals' behavior was video recorded and later scored by an observer blind to the treatment condition. Using a time-sampling procedure every 2 s, each rat was scored blindly as either freezing or active at the instant the sample was taken. Fear was assessed as freezing behavior, defined as behavioral immobility except for movement needed for respiration.

In the experiments involving CFC, after 3 min of exposure to the observation cage, rats received three 1-s footshocks (1 mA intensity) with an inter-trial interval of 60 s. Rats were removed from the conditioning chamber 30 s after the final shock presentation and returned to their home cages. Therefore, the conditioning session lasted 5.5 min. In experiments designed for biochemical analyses that evaluated the impact of CFC in the hippocampal expression of nectins, animals were sacrificed at different times (0.5, 2. 6. 12 or 24 h) after training.

In the experiment performed to compare the impact of exposure to ‘footshock,’ ‘context’ or ‘CFC’ training on nectin expression in the hippocampus, to evaluate the effect of the shock and minimize context exposure (‘shock’ condition), rats were given one 2-s footshock (1 mA) immediately after being introduced in a novel context and were quickly removed and returned to their home cage. To assess the impact of the context (‘context’ condition), rats were allowed to explore the novel context for 5.5 min without receiving any additional stimulation. Animals were sacrificed at 2 h after exposure to footshock, context or CFC.

In the pharmacological experiments that evaluated the impact of anti-nectin-1 infusions in contextual fear memory formation, animals were tested at different times after training; i.e., in one experiment, animals were tested at both 2 and 7 days post-training, while a follow-up study focused directly on the 7 days post-training time point. Testing was performed by placing rats back into the original training context for 8 min, in which no footshock was delivered.

One experiment evaluated the impact of anti-nectin-1 treatment in acoustic fear conditioning. The training protocol was essentially the same as for contextual fear training, with the exception that a tone (85 dB sound lasting 20 s at 1000 Hz) preceded each footshock. Assessment of tone fear memory was performed 7 days post-training by placing animals into a novel context (same cages, but with different walls, floor, illumination and background odor), and after a 2 min and 40 s baseline period, rats were continuously re-exposed to three 20-s tones (with intervals of 40 s) in the absence of shocks.

### Biochemistry

In the experiment that evaluated time-dependent changes in hippocampal nectin expression following fear conditioning, rats were sacrificed at 0.5, 2, 6, 12 or 24 h after training. In the experiment that compared the effect of CFC training with that of exposure to either the shock or the context separately, the animals were sacrificed 2 h after exposure to each of these conditions. In all cases, a control handled-only group was added for comparison. The rats were decapitated, their brains were quickly extracted, and the hippocampi were dissected and stored at −80°C. Synaptoneurosomes and total fractions were prepared as described previously [47] following the method of Hollingsworth et al. [48].

Tissue samples were homogenized in ice-cold homogenization buffer (10 mm HEPES, 1.0 mM EDTA, 2.0 mM EGTA, 0.5 mM dithiothreitol (DTT), 0.1 mM phenylmethanesulfonyl fluoride (PMSF)) containing a freshly added protease and phosphatase inhibitor cocktail (Complete EDTAfree, Roche Diagnostics GmbH, Mannheim, Germany) with an Eppendorf homogenizer. At this stage, aliquots of whole homogenates (total fraction) were taken, solubilized with 1% NP-40, removed of debris with 10 min of 1000 g centrifugation and stored at −80°C for future analysis. The remaining homogenates were passed through two 100 µm-pore nylon mesh filters and then further through two 5 µm-pore filters. Filtered homogenates were centrifuged at 1000 g for 10 min at 4°C. Resultant pellets were resuspended in 100 µL of 1% sodium dodecyl sulfate, boiled for min and stored at −80°C. Whole and synaptoneurosome hippocampal samples were quantified using the detergent-compatible protein assay (Biorad). Equal protein samples were prepared at a concentration of 0.75 µg/mL in 33 mM NaCl, 70 mM Tris–HCl, 1 mM EDTA, 2% (w/v) sodium dodecyl sulfate, 0.01% (w/v) bromophenol blue and 10% glycerol, pH 6.8. Proteins were resolved on 10% polyacrylamide gels and transferred to nitrocellulose membranes. Membranes were blocked for 1 h at room temperature with 5% non-fat dry milk in Tris-buffered saline (TBS)-0.1% Tween-20 buffer. Membranes were then incubated with primary antibodies (nectin-1, 1∶5,000; SCBT H-62 sc-28639; nectin-3, 1∶3,000, Abcam ab63931; pan-actin 1∶20,000, Sigma and GAPDH, 1∶100,000, Abcam 6C5 ab8245) overnight at 4°C. The membranes were washed three times in TBS-0.1% Tween-20 for 10 min and then incubated for 2 h at room temperature with the appropriate secondary horseradish peroxidase-linked antibodies diluted in blocking buffer. Following membrane washing with TBS-0.1% Tween 20 buffer, the immunocomplexes were visualized using a chemiluminescence peroxidase substrate (SuperSignal West Dura Extended Duration Substrate), and immunoreactivity was detected using the ChemiDoc XRS system (Biorad). Densitometry analysis on the bands was calculated using Quantity One 4.2.3 software (Biorad Laboratories AG, Switzerland). Each band was normalized to GAPDH as determined in the corresponding sample. On each gel, at least two naive controls were used, and protein changes were represented as a percentage of the normalized naive value. Protein measurements were performed in the linear range for all immunoblot assays.

### Surgery

Rats subjected to pharmacological experiments were implanted with stainless steel guide cannulas aimed at the dorsal or ventral hippocampus. The rats were anesthetized with a xylazine/ketamine (10/80 mg/kg in a volume of 2 mL/kg) i.p. injection and placed in a stereotaxic apparatus (David Kopf Instruments, Tujunga, CA, USA). Small holes were drilled through the skull for bilateral placement of a stainless steel 22 gauge guide cannulae (Plastics One, Roanoke, VA, USA) fitted with a removable dummy cannula, above the dorsal (3.8 mm posterior, 2.2 mm lateral, and 2.5 mm ventral) or ventral (5.20 mm posterior, 5.0 mm lateral, and 6 mm ventral) hippocampus. Coordinates were based on the atlas of Paxinos and Watson (1986) and are taken from bregma. Cannulae were fixed to the skull with two anchoring screws and dental acrylic (Duralay 2244; Reliance, Worth, IL). After surgery, the animals were housed two per cage, with a separator that allowed visual and odor contact but impeded physical contact in order to preserve cannula implantation. After behavioral experiments animals were sacrificed by i.p. pentobarbital injection and correct cerebral cannulae placement was routinely verified with Evans blue histology.

### Pharmacological experiments involving nectin-1 inhibition

After recovery from surgery, the animals were handled and habituated to the microinfusion procedure. The rats were wrapped in a soft towel, the obturator was removed, and a 28-gauge microinjector (Plastics One, Roanoke, VA, USA) extending 1.0 mm from the tip of the guide cannula attached to polyethylene 50 (cat n. 8010, Phymep, Paris, France) tubing was inserted through the cannula. The distal end of the PE50 tubing was attached to a 10 µL (Hamilton) syringe that was mounted on a microinjection unit (model 5000; David Kopf Instruments, Tujunga, CA, USA).

Infusion of R165 or ACSF was performed immediately after CFC. Microinfusions were performed bilaterally with 1 µl per brain region and were delivered over 2 min. The microinjector remained in place for an additional 1 min following infusion to allow proper diffusion.

The anti-nectin-1 rabbit polyclonal sera R165 used in the studies that interfered with nectin-1 function were described previously [49], [50]. Anti-nectin-1 antibody rabbit polyclonal serum R165 was obtained after immunization with purified human nectin-1 ectodomain HveC(346t), which was produced in insect cells [49]. The R165 serum recognizes the nectin-1 ectodomain and the V-domain. Importantly for our purposes, R165 also recognizes rat nectin-1 found at the surface of neurons [51].

### Statistical analysis

Data are expressed as the mean ± SEM. Behavioral observations were analyzed using an analysis of variance (ANOVA) followed with Bonferroni post-hoc tests when appropriate. Two-sample comparisons were analyzed using the two-tailed Student t-test. Data were considered to be statistically significant when p<0.05. n.s. denotes no significant difference.

## Results

### Contextual fear conditioning leads to a time-dependent increase of nectin-1 hippocampal expression in the synaptoneurosomal fraction

**Figure 1:**
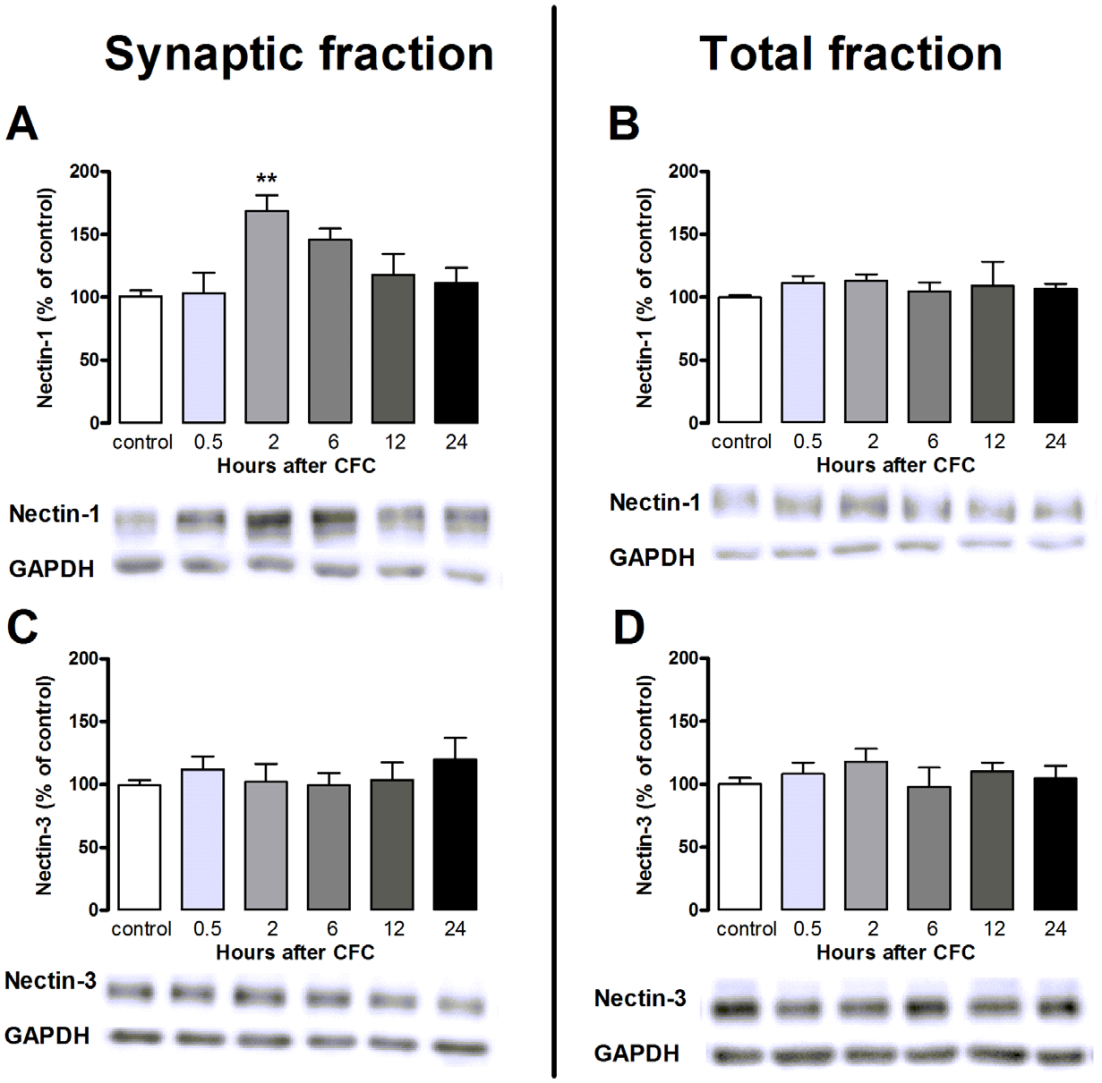
Effect of contextual fear conditioning on nectin-1 (A, B) and nectin-3 (C, D) protein expression in synaptic and total fractions. Synaptic nectin-1 expression was enhanced only at 2 h after CFC (A). Total fraction of nectin-1 (B) as well as synaptic- (C) or total nectin-3 (D) remained unaltered. Error bars represent standard error of the mean (n = 6 animals/group) (** p<0.01 vs. control group indicated by Bonferroni post hoc test).

Levels of hippocampal nectin-1 and nectin-3 were measured in synaptoneurosomal and total fractions at different time points after training rats in the CFC paradigm. Synaptic nectin-1 was transiently increased in the hippocampus at 2 h, but it did not differ from control values at 0.5, 6, 12, or 24 h after training (ANOVA, F_5,30_ = 4.51, p<0.01 and Bonferroni post hoc tests; p<0.01 at 2 h, n.s. for other time-points; Fig. 1A). The increase of nectin-1 was specific for the synaptoneurosomal fraction; protein levels of nectin-1 in the total fraction were not affected by training (ANOVA, F_5,30_ = 0.28, p = 0.92; Fig. 1B). In contrast, hippocampal expression of nectin-3 was not affected by fear conditioning training at any of the time points examined, neither in the synaptoneurosomal nor in the total fractions (synaptoneurosomal fraction: ANOVA, F_5,30_ = 0.71, p = 0.62; total fraction: ANOVA, F_5,30_ = 0.54, p = 0.74, Fig. 1C-D).

### Nectin-1 synaptoneurosomal expression following contextual fear conditioning is increased specifically in the ventral hippocampus

Increased nectin-1 expression observed in the synaptoneurosomal hippocampal fraction 2 h after training in the CFC paradigm might be the result of the integrated CFC experience or induced, independently, by exposure to the key element(s) involved in this training; e.g., the shock and the context. Shock delivery shortly after brief exposure to a new context does not lead to CFC [52] but pre-exposure to the context before shock delivery enables the emergence of CFC [52], [53]. To understand the experimental determinants leading to the hippocampal regulation of nectin-1, we performed an experiment in which, in addition to training animals in the CFC protocol, additional groups of animals were exposed to either the shock with minimal context exposure or to the context without shock stimulation. Samples were taken 2 h after exposure to each of these experimental conditions. In order to evaluate whether the observed increase in nectin-1 expression is confined to a particular hippocampal subdivision, protein expression levels were assessed separately in the ventral and dorsal hippocampus. Nectin-3 analyses were included for comparison.

**Figure 2:**
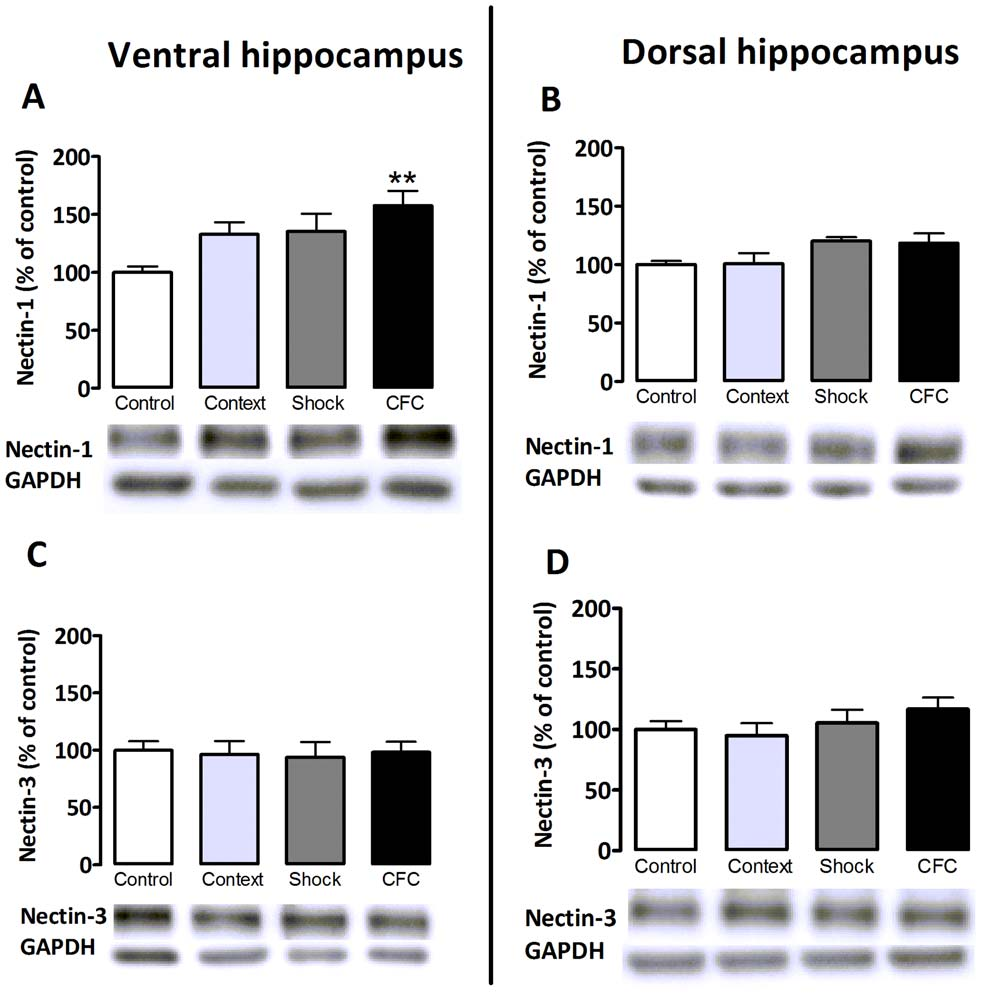
Effect of context, shock and contextual fear conditioning on nectin-1 (A, B) and nectin-3 (C, D) protein expression in synaptic fractions of ventral and dorsal hippocampi. The increase of nectin-1 at 2 h after CFC was not seen after context- or shock exposure alone and was restricted to ventral hippocampus (A). Nectin-1 levels in the dorsal hippocampus were not affected 2 h after shock, context or CFC (B). Nectin-3 levels in both ventral- (C) and dorsal hippocampus (D) were unaffected 2 h after context, shock or CFC. Error bars represent standard error of the mean (n = 7–10 animals/group) (*, p<0.05; ** p<0.01 vs. control group indicated by Bonferroni post hoc test).

In confirmation of the findings obtained in the previous experiment, synaptoneurosomal nectin-1 levels were significantly increased in the ventral hippocampus 2 h after CFC. However, shock or context exposure alone did not significantly affect nectin-1 protein levels in this hippocampal subdivision (Fig. 2A, ventral hippocampus: ANOVA F_3,32_ = 4.94, p<0.006. Bonferroni post hoc tests; p = 0.003 for CFC-group vs. control; p = 0.29 for context-group vs. control and p = 0.21 for shock-group vs. control). In the dorsal hippocampus, levels of synaptic nectin-1 at 2 h after context, shock or exposure to CFC were not affected (Fig. 2B, dorsal hippocampus: ANOVA F_3,31_ = 2.54, p = 0.07). In line with our previous observations, synaptoneurosomal nectin-3 levels remained unchanged at 2 h after CFC training or following exposure to either context or shock only (Fig. 2C-D, ventral hippocampus: ANOVA F_3.34_ = 0.06, p = 0.98; dorsal hippocampus: ANOVA F_3.34_ = 0.99, p = 0.41).

### Administration of a nectin-1 antibody in the ventral hippocampus impairs contextual fear memory

**Figure 3:**
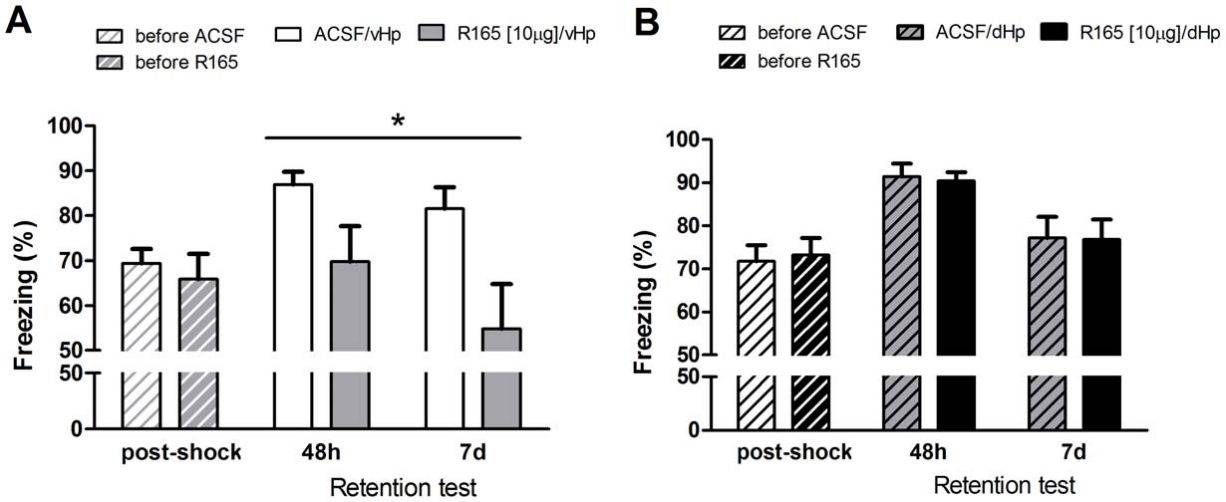
Effect of nectin-1 inhibition on fear memory consolidation. Prior to treatment, there was no difference in the freezing response during CFC. Inhibition of nectin-1 by R165 infusion in the ventral hippocampus immediately after CFC reduced freezing as measured at 2 and 7 d after contextual fear training (A). This effect was not seen for the dorsal hippocampus (B). Error bars represent standard error of the mean (n = 7 animals/group) (n.s., no significant difference; *, p<0.05: treatment effect R165 vs. control indicated by two-way ANOVA).

Given the increase in nectin-1 synaptoneurosomal expression that we found in the ventral hippocampus following training in the CFC task in the previous experiments, we aimed to investigate the involvement of nectin-1 in memory consolidation. For this purpose, the anti-nectin-1 antibody R165 (see Materials and Methods) was infused in the ventral hippocampus immediately after training. As expected, before infusion, the groups did not differ in the freezing response resulting from the shock during contextual fear training (Fig. 3A, ventral-hippocampus t-test t = 0.55, df = 12, p = 0.59, Fig. 3B, dorsal hippocampus t-test t = 0.23, df = 12, p = 0.82). Next, the impact of anti-nectin-1 antibody R165 in the ventral hippocampus on contextual fear memory was tested 2 and 7 days after training. Treatment with R165 in the ventral hippocampus reduced contextual freezing across the different testing times (Fig. 3A; two-way ANOVA with repeated measures: main effect of treatment, F_1,12_ = 5.99, p = 0.03; no interaction effect, F_1,12_ = 1.55, p = 0.24). When these animals were tested the following day in a novel context (thus at 72 h and 8 d after CFC-training), freezing-levels did not differ between vehicle and R165-treated animals (72 h, vehicle: 20.6+/−6.7 s; R165: 19.1+/−4.9 s, t-test, t = 0.18, df = 11, p = 0.86 and at 8 d, vehicle: 21.2+/−5.4 s; R165: 17.5+/−5.4 s, t-test, t = 0.48, df = 12, p = 0.64), excluding the possibility that the treatment would be inducing changes in context generalization.

Contrasting to the effects seen with R165 treatment in the ventral hippocampus, infusion of the nectin-1 antibody in the dorsal hippocampus did not affect subsequent contextual fear memory tested at 2 and 7 days post-training (Fig. 3B; two-way ANOVA with repeated measures: main effect of treatment, F_1,10_ = 0.02, p = 0.88).

**Figure 4:**
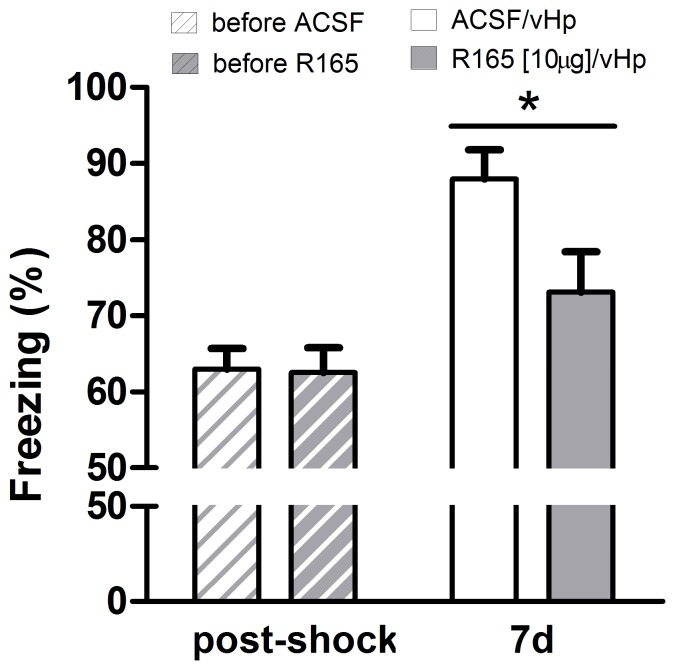
Effect of nectin-1 on fear memory consolidation tested 7 d after training. Prior to treatment, there was no difference in the freezing response during CFC. Inhibition of nectin-1 by R165 infusion in the ventral hippocampus immediately after CFC reduced freezing when tested at 7d later (n = 7 animals/group) (n.s., no significant difference; *, p<0.05 vs. control group, two-tailed Student t-test).

As a stronger effect of the treatment was observed at 7 days post-training, it is possible that the global effect was due to repeated testing in the same animals and, thus, to an interaction between the drug treatment and potential extinction mechanisms. To evaluate whether R165 administration would be enough to induce a significant reduction in freezing levels when animals were tested 7 days post-training, a follow-up experiment was performed in which animals were infused with R165 in the ventral hippocampus after training and were tested only at this latter time point. We first confirmed that prior to infusion, the groups did not differ in the amount of freezing upon shock (Fig. 4, t-test t = 0.13, df = 12, p = 0.90). However, at 7 days after training, the animals treated with R165 again showed impaired contextual fear memory (Fig. 4, t-test t = 2.30, df = 12, p = 0.04). This experiment further confirmed that interfering with nectin-1 in the ventral hippocampus immediately after training reduces the strength of the subsequent contextual fear memory formed in a long-lasting manner.

To further assay for the specificity of the results observed in CFC when the R165 antibody was administered into the ventral hippocampus, we performed another experiment to investigate whether the same treatment would influence the consolidation of an acoustic fear memory. Rats were trained in the acoustic fear conditioning protocol and then immediately infused with the R165 antibody into the ventral hippocampus. When tested for the tone memory at 7 days post-training, no effect of the treatment was observed (freezing response to tone, vehicle: 98.0+/−1.3 s; R165: 97.6+/−2.0 s, t-test: t = 0.19, df = 11, p = 0.86).

### Administration of the nectin-1 antibody into the ventral hippocampus does not affect anxiety-like behavior or locomotor activity

**Figure 5:**
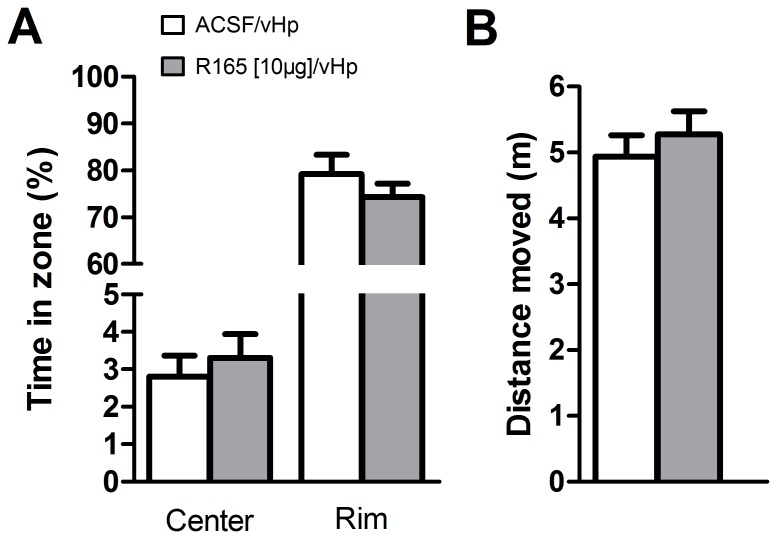
Effects of nectin-1 inhibition in the ventral hippocampus on anxiety-like behavior (A) and locomotor activity (B). Inhibition of nectin-1 by infusion of R165 into the ventral hippocampus did not affect time spent in the center, the rim area of the open field as measured 7 d later (A). In addition, locomotor activity in the open field was also not affected by R165 infusion 7 d before (B). Error bars represent standard error of the mean (n = 14 for ACSF-treated animals, n = 19 for R165-treated animals).

The previous experiment indicated that when delivered into the ventral hippocampus, the nectin-1 antibody impaired the formation of contextual fear memory. However, it could be argued that the reduced freezing observed at testing could be due to potential non-specific memory effects, such as changes in anxiety-like behavior and/or locomotor activity. To investigate these possibilities, the animals were infused with either vehicle or the nectin-1 antibody into the ventral hippocampus and tested 7 d later with the open field test (∅ 1 m for 10 min at 7 lux). Time spent in the center or in the outer zone of the open field was not affected by the infusion of R165 (Fig. 5A; t-test: center: t = 0.57, df = 31, p = 0.57; rim: t = 1.01, df = 31, p = 0.32), indicating a lack of influence of this treatment on anxiety-like behavior. In addition, locomotor activity did not differ between treatment groups, as indicated by the equal distance moved throughout the open field session (Fig. 5B; t-test t = 0.69, df = 31, p = 0.49).

## Discussion

This study identified a time-dependent upregulation of nectin-1 expression in the synaptoneurosomal, but not total, compartment in the ventral hippocampus following CFC in rats. In contrast, nectin-3 levels were not affected by CFC. The increase of nectin-1 was specifically found in the ventral part of the hippocampus at the 2 h post-training time point, with no changes observed in the dorsal hippocampus at any of the post-training times examined (0.5, 2, 6, 12 and 24 h). In line with these findings, interfering with nectin-1 functioning by injecting a specific nectin-1 antibody in the ventral hippocampus immediately after CFC training interfered with memory for the context, whereas the same treatment given in the dorsal hippocampus did not elicit an effect. Overall, these results support a role for nectin-1 in the ventral hippocampus in contextual fear-conditioned memory.

Evidence has been presented for two time periods of protein synthesis that occur in the hippocampus and are required for memory consolidation of fear-motivated learning: the first at about the time of training and the second 3–6 h post-training [54]. Although the former would be compatible with the increased expression of nectin-1 that we observed in the synaptoneurosomal fraction of the ventral hippocampus, the fact that no changes were detected in the whole homogenate fraction makes it unlikely that the observed effect resulted from the *de novo* synthesis of nectin-1. However, although our results would suggest that the observed effects were due to the activity-dependent recruitment of nectin-1 toward the perisynaptic region, we cannot discard the involvement of protein synthesis in the process (e.g., increased synaptoneurosomal expression of nectin-1 could be linked to the training-induced synthesis of an interacting carrier or recruiting molecule).

Neuronal nectin-1 may bind functionally to nectin-1 to itself, to nectin-3 or to the fibroblast growth factor receptor (FGFR) [4], [55]. Nectin-3 and nectin-1 share a binding site on the first Immunoglobulin-like domain (V-domain) of nectin-1 [56], [57] and promote cellular and synaptic adhesion. In contrast, FGFR interacts with the third Ig like domain (C domain) of nectin-1, which results in neurite outgrowth and neuron survival ex vivo [55]. The polyclonal serum R165 contains antibodies to epitopes in each nectin-1 domain and thus may interfere with binding of any of the three ligands thereby affecting adhesion and signaling. In the context of synaptic adhesion, it is unclear whether the antibody can access nectin-1 when it is already engaged with a ligand and disrupt established intercellular interactions in vivo. However, nectin-1 antibodies can prevent ligand binding and the establishment of interactions leading to cell adhesion [58]. Interestingly, CFC leads to an increase of nectin-1 in the synaptoneurosomal fraction rather than in the total neuronal fraction (Fig. 1). This suggests that a ligand-free nectin-1 is recruited to the synapse where it is retained by trans-interacting with a ligand, possibly nectin-3. In this adhesion model, the antibody may interfere with recruitment and/or ligand binding, thereby altering the adhesive or signaling function of nectin-1 at synapses. In the context of FGFR signaling, the antiserum may prevent nectin-1 binding to FGFR, which activation by NCAM has been shown to promote memory consolidation and synapse formation [45]. More specific targeting of either function of nectin-1 is needed to identify the mechanism of action of nectin-1 in CFC which will improve our understanding of the molecular basis of contextual fear memory.

A key question to address is the temporal dynamics of the observed effects. In fact, we should note that a typical feature revealed by studies that addressed the involvement of cell adhesion molecules in memory consolidation is the transient nature of their involvement. The intracerebral infusion of antibodies against specific cell adhesion molecules (e.g., integrins [59], [60], NCAM [61], [62], PSA-NCAM [63]) or their interacting partners (e.g., cellular prion protein [64]) has proved to be a useful tool to demonstrate a role for these molecules in memory consolidation. For example, the intracerebroventricular infusion of an antibody against NCAM was shown to inhibit the consolidation of a passive avoidance task when administered in the 6–8 h post-training period, but not at other time points [61]. PSA-NCAM was found to be transiently increased in the hippocampus at about 12–24 hours post-training, but not at earlier or later time points [65], [66], [67]. Accordingly, the intracerebroventricular infusion of an antibody against PSA-NCAM at 10 h post-training in a passive avoidance task induced subsequent amnesia for the learned response [63]. These results have been typically interpreted to reflect late time windows of involvement of these molecules in the mechanisms of memory consolidation. Our results, which showed a transient increase of nectin-1 in the synaptoneurosomal fraction in the ventral, but not dorsal, hippocampus and an amnestic effect for the posttraining administration of a nectin-1 antibody when infused in the ventral, but not dorsal, hippocampus, support the involvement of this adhesion molecule in the early post-training mechanisms that act in the ventral hippocampal subfield to promote the consolidation of the contextual fear memory.

Although lesion and pharmacological studies have supported a role for both ventral and dorsal hippocampal subfields in contextual fear memory formation [24], [25], [27], [28], the underlying cellular and molecular mechanisms that participate in the processing of contextual fear memory in each subfield are largely unknown. An important reason for this lack of knowledge is that a great majority of studies that have identified upstream and downstream protein synthesis mechanisms have overlooked potential differences in the different hippocampal parts [41], [68], [69]. Recent evidence from studies that dissociate changes in each hippocampal part supports the existence of both common and subfield-specific mechanisms. Plasticity within both the dorsal and ventral hippocampus has been shown to be required for the acquisition and maintenance of contextual fear-conditioning memory. Thus, the Arc gene and protein were found to increase in both the dorsal and ventral hippocampus after CFC in rats, and Arc knockdown using antisense oligodeoxynucleotide administration in either of these hippocampal subfields effectively impaired contextual memory formation [70]. The involvement of Arc in each hippocampal region was found to involve activation of NMDA receptors [70], [71]. However, the existence of region-specific mechanisms is supported by a number of studies. For example, nicotine facilitated CFC when infused in the dorsal hippocampus but impaired CFC when given in the ventral hippocampus [72]. Evidence was provided for the specific involvement of PSA-NCAM in the dorsal, but not ventral, hippocampus, as indicated by a specific training-induced increase in the ventral part and by experiments involving the removal of PSA-NCAM, effectively interfering with contextual fear memory formation when given in the dorsal, but not ventral, hippocampus [43]. Our findings clearly support the existence of hippocampal subregion-specific mechanisms in the formation of contextual fear memory by highlighting the importance of nectin-1 during the early post-training period in the ventral but not dorsal hippocampus.

Only the combination of context exposure and shock (i.e., the putative CFC protocol) led to increased levels of synaptic nectin-1 in the ventral hippocampus, while context or shock exposure alone only resulted in non-significant increases. The fact that context exposure on its own did not induce the same effect as the full fear conditioning experience is at odds with the molecular changes observed in the hippocampus in previous studies using a similar experimental protocol. Thus, although the shock alone was not followed by changes in the parameters examined [73], exposing animals just to the context was enough to induce similar activation in the hippocampal expression of the immediate early genes c-fos and Arc [74] and in dorsal hippocampus PSA-NCAM expression [43] as that found for the full CFC experience. The fact that nectin-1 molecular changes induced by CFC in the ventral hippocampus were not reproduced by only context exposure might be indicative of the type of computations that occur in this hippocampal compartment as opposed to those relying in the dorsal component. Given the close interconnections between the ventral hippocampus and the amygdala [18], this hippocampal part has been considered to be a gateway that transfers contextual information between the dorsal hippocampus and the amygdala [75]. The rapid involvement of nectin-1 in the post-training period (as opposed to late post-training windows of involvement reported for other adhesion molecules; see Introduction and Discussion above) only when converging context and shock information is processed would be in line with the role of the ventral hippocampus in linking contextual information processed by the dorsal hippocampus with emotional information processed by the amygdala [18], [19] in the aftermath of the conditioning experience. Importantly, the specificity of the involvement of nectin-1 in the ventral hippocampus in contextual fear memory was further supported by a follow-up experiment in which memory for a tone was not impaired by the post-training administration of the nectin-1 antibody in the ventral hippocampus following training in the auditory fear conditioning task.

The fact that most manipulations assessing the role of ventral hippocampus involved lesions or pharmacological manipulations that also resulted in increased anxiety and/or locomotor activity [24], [24], [25], [27,76] has complicated the understanding of the functional role of this hippocampal region. In our study, in order to exclude the possibility that the effects elicited by the infusion of the antibody targeting nectin-1 on freezing behavior were confounded by anxiety or locomotor activity, rats were infused with the antibody in the ventral hippocampus and tested in the open field. The treatment with the nectin-1 antibody did not affect the time spent in the center or rim of the open field (but note that the open field is not a test specific for anxiety and time spent in the center by control animals was low which could have made difficult the possibility to detect a further decrease in this measure by the treatment), nor did this alter animals' locomotor activity. Importantly, the lack of effect of blockade of nectin-1 function in the ventral hippocampus in anxiety or locomotion does not imply a lack of involvement of this hippocampal subdivision in these previously proposed functions [77], [78], [79]. Our findings only suggest that nectin-1 seems not to be required for the alterations in the parameters that were formerly described following specific lesions to the ventral hippocampus.

In addition to a potential role on neural plasticity (see introduction), Nectin-1 is also the main receptor used by herpes simplex virus (HSV) to infect and spread between neurons [51], [80]. Nectin-1 is highly expressed in murine hippocampus and other areas susceptible to HSV infection [81], [82]. In acute HSV encephalitis (HSE) the virus causes necrotizing lesions in the temporal lobes and limbic structures [83]. HSE lesions are also commonly found in the hippocampus and amygdala [83] and may result in impaired cognitive abilities [84], [85], [86]. Rats which recovered from experimental HSE have impaired spatial recognition memory in the absence of residual visible neuropathological damage [84]. Since HSV causes nectin-1 downregulation [87], [88], one may speculate that, in addition to HSV neuropathy, transient decrease of synaptic nectin-1 may affect long term cognitive ability.

In summary, we have presented compelling evidence for a role of nectin-1 in the ventral hippocampus in contextual fear memory consolidation. Our results highlight this molecule as a potential novel player in the specific mechanisms whereby the ventral hippocampus connects emotional and contextual information from the amygdala and dorsal hippocampus, respectively, and open new venues to explore nectin-1 as a potential novel target for the development of treatments to psychopathological alterations that are linked to the impaired contextualization of emotions.

## References

[1] Takai Y, Shimizu K, Ohtsuka T (2003) The roles of cadherins and nectins in interneuronal synapse formation. Curr Opin Neurobiol 13: 520–6. 14630213 10.1016/j.conb.2003.09.003

[2] Takai Y, Miyoshi J, Ikeda W, Ogita H (2008) Nectins and nectin-like molecules: roles in contact inhibition of cell movement and proliferation. Nat Rev Mol Cell Biol 9: 603–15. 18648374 10.1038/nrm2457

[3] Togashi H, Miyoshi J, Honda T, Sakisaka T, Takai Y, et al (2006) Interneurite affinity is regulated by heterophilic nectin interactions in concert with the cadherin machinery. J Cell Biol 174: 141–51. 16801389 10.1083/jcb.200601089 PMC2064171

[4] Mizoguchi A, Nakanishi H, Kimura K, Matsubara K, Ozaki-Kuroda K, et al (2002) Nectin: an adhesion molecule involved in formation of synapses. J Cell Biol 156: 555–65. 11827984 10.1083/jcb.200103113 PMC2173327

[5] Honda T, Sakisaka T, Yamada T, Kumazawa N, Hoshino T, et al (2006) Involvement of nectins in the formation of puncta adherentia junctions and the mossy fiber trajectory in the mouse hippocampus. Mol Cell Neurosci 31: 315–25. 16300961 10.1016/j.mcn.2005.10.002

[6] Lim ST, Chang A, Giuliano RE, Federoff HJ (2012) Ectodomain shedding of nectin-1 regulates the maintenance of dendritic spine density. J Neurochem 120: 741–51. 22118475 10.1111/j.1471-4159.2011.07592.x

[7] Horváth S, Prandovsky E, Kis Z, Krummenacher C, Eisenberg RJ, et al (2006) Spatiotemporal changes of the herpes simplex virus entry receptor nectin-1 in murine brain during postnatal development. J Neurovirol 12: 161–70. 16877297 10.1080/13550280600760594

[8] Okabe N, Shimizu K, Ozaki-Kuroda K, Nakanishi H, Morimoto K, et al (2004) Contacts between the commissural axons and the floor plate cells are mediated by nectins. Dev Biol 273: 244–56. 15328010 10.1016/j.ydbio.2004.05.034

[9] Martin SJ, Grimwood PD, Morris RG (2000) Synaptic plasticity and memory: an evaluation of the hypothesis. Annu Rev Neurosci 23: 649–711. 10845078 10.1146/annurev.neuro.23.1.649

[10] Shapiro M (2001) Plasticity, hippocampal place cells, and cognitive maps. Arch Neurol 58: 874–81. 11405801 10.1001/archneur.58.6.874

[11] O'Sullivan NC, McGettigan PA, Sheridan GK, Pickering M, Conboy L, et al (2007) Temporal change in gene expression in the rat dentate gyrus following passive avoidance learning. J Neurochem 101: 1085–98. 17298388 10.1111/j.1471-4159.2006.04418.x

[12] Garcia R, Tocco G, Baudry M, Thompson RF (1998) Exposure to a conditioned aversive environment interferes with long-term potentiation induction in the fimbria-CA3 pathway. Neuroscience 82: 139–45. 9483510 10.1016/s0306-4522(97)00285-6

[13] Restivo L, Vetere G, Bontempi B, Ammassari-Teule M (2009) The formation of recent and remote memory is associated with time-dependent formation of dendritic spines in the hippocampus and anterior cingulate cortex. J Neurosci 29: 8206–14. 19553460 10.1523/JNEUROSCI.0966-09.2009 PMC6666032

[14] Motanis H, Maroun M (2010) Exposure to a novel context following contextual fear conditioning enhances the induction of hippocampal long-term potentiation. Eur J Neurosci 32: 840–6. 20649905 10.1111/j.1460-9568.2010.07334.x

[15] Kim JJ, Fanselow MS (1992) Modality-specific retrograde amnesia of fear. Science 256: 675–7. 1585183 10.1126/science.1585183

[16] Phillips RG, LeDoux JE (1992) Differential contribution of amygdala and hippocampus to cued and contextual fear conditioning. Behav Neurosci 106: 274–85. 1590953 10.1037//0735-7044.106.2.274

[17] Corcoran KA, Maren S (2001) Hippocampal inactivation disrupts contextual retrieval of fear memory after extinction. J Neurosci 21: 1720–6. 11222661 10.1523/JNEUROSCI.21-05-01720.2001 PMC6762930

[18] Moser MB, Moser EI (1998) Functional differentiation in the hippocampus. Hippocampus 8: 608–619. 9882018 10.1002/(SICI)1098-1063(1998)8:6<608::AID-HIPO3>3.0.CO;2-7

[19] Fanselow MS, Dong HW (2010) Are the dorsal and ventral hippocampus functionally distinct structures? Neuron 65: 7–19. 20152109 10.1016/j.neuron.2009.11.031 PMC2822727

[20] Hunsaker MR, Kesner RP (2008) Dissociations across the dorsal-ventral axis of CA3 and CA1 for encoding and retrieval of contextual and auditory-cued fear. Neurobiol Learn Mem 89: 61–9. 17931914 10.1016/j.nlm.2007.08.016 PMC2675280

[21] Gafford GM, Parsons RG, Helmstetter FJ (2011) Consolidation and reconsolidation of contextual fear memory requires mammalian target of rapamycin-dependent translation in the dorsal hippocampus. Neuroscience 182: 98–104. 21439355 10.1016/j.neuroscience.2011.03.023 PMC3087706

[22] Mitsushima D, Ishihara K, Sano A, Kessels HW, Takahashi T (2011) Contextual learning requires synaptic AMPA receptor delivery in the hippocampus. Proc Natl Acad Sci U S A 108: 12503–8. 21746893 10.1073/pnas.1104558108 PMC3145714

[23] Anagnostaras SG, Gale GD, Fanselow MS (2001) Hippocampus and contextual fear conditioning: recent controversies and advances. Hippocampus 11: 8–17. 11261775 10.1002/1098-1063(2001)11:1<8::AID-HIPO1015>3.0.CO;2-7

[24] Maren S (1999) Neurotoxic or electrolytic lesions of the ventral subiculum produce deficits in the acquisition and expression of Pavlovian fear conditioning in rats. Behav Neurosci 113: 283–90. 10357453 10.1037//0735-7044.113.2.283

[25] Richmond MA, Yee BK, Pouzet B, Veenman L, Rawlins JN, et al (1999) Dissociating context and space within the hippocampus: effects of complete, dorsal, and ventral excitotoxic hippocampal lesions on conditioned freezing and spatial learning. Behav Neurosci 113: 1189–203. 10636298 10.1037/0735-7044.113.6.1189

[26] Bast T, Zhang WN, Feldon J (2001) Hyperactivity, decreased startle reactivity, and disrupted prepulse inhibition following disinhibition of the rat ventral hippocampus by the GABA(A) receptor antagonist picrotoxin. Psychopharmacology (Berl) 156: 225–33. 11549225 10.1007/s002130100775

[27] Zhang WN, Bast T, Feldon J (2001) The ventral hippocampus and fear conditioning in rats: different anterograde amnesias of fear after infusion of N-methyl-D-aspartate or its noncompetitive antagonist MK-801 into the ventral hippocampus. Behav Brain Res 126: 159–74. 11704261 10.1016/s0166-4328(01)00256-x

[28] Esclassan F, Coutureau E, Di Scala G, Marchand AR (2009) Differential contribution of dorsal and ventral hippocampus to trace and delay fear conditioning. Hippocampus 19: 33–44. 18683846 10.1002/hipo.20473

[29] Trivedi MA, Coover GD (2004) Lesions of the ventral hippocampus, but not the dorsal hippocampus, impair conditioned fear expression and inhibitory avoidance on the elevated T-maze. Neurobiol Learn Mem 81: 172–84. 15082019 10.1016/j.nlm.2004.02.005

[30] Hobin JA, Ji J, Maren S (2006) Ventral hippocampal muscimol disrupts context-specific fear memory retrieval after extinction in rats. Hippocampus 16: 174–82. 16358312 10.1002/hipo.20144

[31] Sutherland RJ, O'Brien J, Lehmann H (2008) Absence of systems consolidation of fear memories after dorsal, ventral, or complete hippocampal damage. Hippocampus 18: 710–8. 18446823 10.1002/hipo.20431

[32] Murphy KJ, Regan CM (1998) Contributions of cell adhesion molecules to altered synaptic weightings during memory consolidation. Neurobiol Learn Mem 70: 73–81. 9753588 10.1006/nlme.1998.3839

[33] Dityatev A, Bukalo O, Schachner M (2008) Modulation of synaptic transmission and plasticity by cell adhesion and repulsion molecules. Neuron Glia Biol 4: 197–209. 19674506 10.1017/S1740925X09990111

[34] Kiss JZ, Muller D (2001) Contribution of the neural cell adhesion molecule to neuronal and synaptic plasticity. Rev Neurosci 12: 297–310. 11783716 10.1515/revneuro.2001.12.4.297

[35] Sandi C (2004) Stress, cognitive impairment and cell adhesion molecules. Nat Rev Neurosci 5: 917–30. 15550947 10.1038/nrn1555

[36] Venero C, Herrero AI, Touyarot K, Cambon K, López-Fernández MA, et al (2006) Hippocampal up-regulation of NCAM expression and polysialylation plays a key role on spatial memory. Eur J Neurosci 23: 1585–95. 16553622 10.1111/j.1460-9568.2006.04663.x

[37] Bisaz R, Conboy L, Sandi C (2009) Learning under stress: a role for the neural cell adhesion molecule NCAM. Neurobiol Learn Mem 91: 333–42. 19041949 10.1016/j.nlm.2008.11.003

[38] Stork O, Welzl H, Wolfer D, Schuster T, Mantei N, et al (2000) Recovery of emotional behaviour in neural cell adhesion molecule (NCAM) null mutant mice through transgenic expression of NCAM180. Eur J Neurosci 12: 3291–306. 10998113 10.1046/j.1460-9568.2000.00197.x

[39] Albrecht A, Bergado-Acosta JR, Pape HC, Stork O (2010) Role of the neural cell adhesion molecule (NCAM) in amygdalo-hippocampal interactions and salience determination of contextual fear memory. Int J Neuropsychopharmacol 13: 661–74. 20003620 10.1017/S1461145709991106

[40] Bisaz R, Sandi C (2010) The role of NCAM in auditory fear conditioning and its modulation by stress: a focus on the amygdala. Genes Brain Behav 9: 353–64. 20059553 10.1111/j.1601-183X.2010.00563.x

[41] Merino JJ, Cordero MI, Sandi C (2000) Regulation of hippocampal cell adhesion molecules NCAM and L1 by contextual fear conditioning is dependent upon time and stressor intensity. Eur J Neurosci 12: 3283–90. 10998112 10.1046/j.1460-9568.2000.00191.x

[42] Sandi C, Merino JJ, Cordero MI, Kruyt ND, Murphy KJ, et al (2003) Modulation of hippocampal NCAM polysialylation and spatial memory consolidation by fear conditioning. Biol Psychiatry 54: 599–607. 13129654 10.1016/s0006-3223(03)00182-3

[43] Lopez-Fernandez MA, Montaron MF, Varea E, Rougon G, Venero C, et al (2007) Upregulation of polysialylated neural cell adhesion molecule in the dorsal hippocampus after contextual fear conditioning is involved in long-term memory formation. J Neurosci 27: 4552–61. 17460068 10.1523/JNEUROSCI.0396-07.2007 PMC6673006

[44] Cambon K, Venero C, Berezin V, Bock E, Sandi C (2003) Post-training administration of a synthetic peptide ligand of the neural cell adhesion molecule, C3d, attenuates long-term expression of contextual fear conditioning. Neuroscience 122: 183–91. 14596859 10.1016/s0306-4522(03)00597-9

[45] Cambon K, Hansen SM, Venero C, Herrero AI, Skibo G, et al (2004) A synthetic neural cell adhesion molecule mimetic peptide promotes synaptogenesis enhances presynaptic function, and facilitates memory consolidation. J Neurosci 24: 4197–204. 15115815 10.1523/JNEUROSCI.0436-04.2004 PMC6729275

[46] Wang XD, Chen Y, Wolf M, Wagner KV, Liebl C, et al (2011) Forebrain CRHR1 deficiency attenuates chronic stress-induced cognitive deficits and dendritic remodeling. Neurobiol Dis 42: 300–10. 21296667 10.1016/j.nbd.2011.01.020 PMC3200197

[47] Conboy L, Sandi C (2010) Stress at learning facilitates memory formation by regulating AMPA receptor trafficking through a glucocorticoid action. Neuropsychopharmacology 35: 674–85. 19890264 10.1038/npp.2009.172 PMC3055605

[48] Hollingsworth EB, McNeal ET, Burton JL, Williams RJ, Daly JW, et al (1985) Biochemical characterization of a filtered synaptoneurosome preparation from guinea pig cerebral cortex: cyclic adenosine 3':5'-monophosphate-generating systems, receptors, and enzymes. J Neurosci 5: 2240–53. 2991484 10.1523/JNEUROSCI.05-08-02240.1985 PMC6565304

[49] Krummenacher C, Nicola AV, Whitbeck JC, Lou H, Hou W, et al (1998) Herpes simplex virus glycoprotein D can bind to poliovirus receptor-related protein 1 or herpesvirus entry mediator, two structurally unrelated mediators of virus entry. J Virol 72: 7064–74. 9696799 10.1128/jvi.72.9.7064-7074.1998 PMC109927

[50] Shukla D, Dal Canto MC, Rowe CL, Spear PG (2000) Striking similarity of murine nectin-1alpha to human nectin-1alpha (HveC) in sequence and activity as a glycoprotein D receptor for alphaherpesvirus entry. J Virol 74: 11773–81. 11090177 10.1128/jvi.74.24.11773-11781.2000 PMC112460

[51] Richart SM, Simpson SA, Krummenacher C, Whitbeck JC, Pizer LI, et al (2003) Entry of herpes simplex virus type 1 into primary sensory neurons in vitro is mediated by Nectin-1/HveC. J Virol 77: 3307–11. 12584355 10.1128/JVI.77.5.3307-3311.2003 PMC149788

[52] Fanselow MS (2000) Contextual fear, gestalt memories, and the hippocampus. Behav Brain Res 110: 73–81. 10802305 10.1016/s0166-4328(99)00186-2

[53] Rudy JW, O'Reilly RC (2001) Conjunctive representations, the hippocampus, and contextual fear conditioning. Cogn Affect Behav Neurosci 1: 66–82. 12467104 10.3758/cabn.1.1.66

[54] Igaz LM, Vianna MR, Medina JH, Izquierdo I (2002) Two time periods of hippocampal mRNA synthesis are required for memory consolidation of fear-motivated learning. J Neurosci 22: 6781–9. 12151558 10.1523/JNEUROSCI.22-15-06781.2002 PMC6758123

[55] Bojesen KB, Clausen O, Rohde K, Christensen C, Zhang L, et al (2012) Nectin-1 binds and signals through the fibroblast growth factor receptor. J Biol Chem 287: 37420–33. 22955284 10.1074/jbc.M112.345215 PMC3481338

[56] Fabre S, Reymond N, Cocchi F, Menotti L, Dubreuil P, et al (2002) Prominent role of the Ig-like V domain in trans-interactions of nectins. Nectin3 and nectin 4 bind to the predicted C-C'-C"-D beta- strands of the nectin1 V domain. J Biol Chem 277: 27006–13. 12011057 10.1074/jbc.M203228200

[57] Di Giovine P, Settembre EC, Bhargava AK, Luftig MA, Lou H, et al (2011) Structure of herpes simplex virus glycoprotein D bound to the human receptor nectin-1. PLoS Pathog 7: e1002277. 21980294 10.1371/journal.ppat.1002277 PMC3182920

[58] Krummenacher C, Baribaud I, Sanzo JF, Cohen GH, Eisenberg RJ (2002) Effects of herpes simplex virus on structure and function of nectin- 1/HveC. J Virol 76: 2424–33. 11836420 10.1128/jvi.76.5.2424-2433.2002 PMC153823

[59] Chang HP, Ma YL, Wan FJ, Tsai LY, Lindberg FP, et al (2001) Functional blocking of integrin-associated protein impairs memory retention and decreases glutamate release from the hippocampus. Neuroscience 102: 289–96. 11166115 10.1016/s0306-4522(00)00478-4

[60] Niedringhaus M, Chen X, Dzakpasu R, Conant K (2012) MMPs and soluble ICAM-5 increase neuronal excitability within in vitro networks of hippocampal neurons. PLoS One 7: e42631 doi:10.1371/journal.pone.0042631 22912716 10.1371/journal.pone.0042631 PMC3418258

[61] Doyle E, Nolan PM, Bell R, Regan CM (1992) Intraventricular infusions of anti-neural cell adhesion molecules in a discrete posttraining period impair consolidation of a passive avoidance response in the rat. J Neurochem 59: 1570–3. 1402906 10.1111/j.1471-4159.1992.tb08477.x

[62] Rønn LC, Bock E, Linnemann D, Jahnsen H (1995) NCAM-antibodies modulate induction of long-term potentiation in rat hippocampal CA1. Brain Res 677: 145–51. 7606459 10.1016/0006-8993(95)00147-i

[63] Seymour CM, Foley AG, Murphy KJ, Regan CM (2008) Intraventricular infusions of anti-NCAM PSA impair the process of consolidation of both avoidance conditioning and spatial learning paradigms in Wistar rats. Neuroscience 157: 813–20. 18948173 10.1016/j.neuroscience.2008.09.041

[64] Coitinho AS, Lopes MH, Hajj GN, Rossato JI, Freitas AR, et al (2007) Short-term memory formation and long-term memory consolidation are enhanced by cellular prion association to stress-inducible protein 1. Neurobiol Dis 26: 282–90. 17329112 10.1016/j.nbd.2007.01.005

[65] Fox GB, O'Connell AW, Murphy KJ, Regan CM (1995) Memory consolidation induces a transient and time-dependent increase in the frequency of neural cell adhesion molecule polysialylated cells in the adult rat hippocampus. J Neurochem 65: 2796–9. 7595580 10.1046/j.1471-4159.1995.65062796.x

[66] O'Connell AW, Fox GB, Barry T, Murphy KJ, Fichera G, et al (1997) Spatial learning activates neural cell adhesion molecule polysialylation in a corticohippocampal pathway within the medial temporal lobe. J Neurochem 68: 2538–46. 9166750 10.1046/j.1471-4159.1997.68062538.x

[67] Foley AG, Hedigan K, Roullet P, Moricard Y, Murphy KJ, et al (2003) Consolidation of memory for odour-reward association requires transient polysialylation of the neural cell adhesion molecule in the rat hippocampal dentate gyrus. J Neurosci Res 74: 570–6. 14598301 10.1002/jnr.10758

[68] Huff NC, Rudy JW (2004) The amygdala modulates hippocampus-dependent context memory formation and stores cue-shock associations. Behav Neurosci 118: 53–62. 14979782 10.1037/0735-7044.118.1.53

[69] Frankland PW, Josselyn SA, Anagnostaras SG, Kogan JH, Takahashi E, et al (2004) Consolidation of CS and US representations in associative fear conditioning. Hippocampus 14: 557–69. 15301434 10.1002/hipo.10208

[70] Czerniawski J, Ree F, Chia C, Ramamoorthi K, Kumata Y, et al (2011) The importance of having Arc: expression of the immediate-early gene Arc is required for hippocampus-dependent fear conditioning and blocked by NMDA receptor antagonism. J Neurosci 31: 11200–7. 21813681 10.1523/JNEUROSCI.2211-11.2011 PMC6623359

[71] Czerniawski J, Ree F, Chia C, Otto T (2012) Dorsal versus ventral hippocampal contributions to trace and contextual conditioning: differential effects of regionally selective NMDA receptor antagonism on acquisition and expression. Hippocampus 22: 1528–39. 22180082 10.1002/hipo.20992

[72] Kenney JW, Raybuck JD, Gould TJ (2012) Nicotinic receptors in the dorsal and ventral hippocampus differentially modulate contextual fear conditioning. Hippocampus 22: 1681–90. 22271264 10.1002/hipo.22003 PMC3343189

[73] Hall J, Thomas KL, Everitt BJ (2000) Rapid and selective induction of BDNF expression in the hippocampus during contextual learning. Nat Neurosci 3: 533–5. 10816306 10.1038/75698

[74] Huff NC, Frank M, Wright-Hardesty K, Sprunger D, Matus-Amat P, et al (2006) Amygdala regulation of immediate-early gene expression in the hippocampus induced by contextual fear conditioning. J Neurosci 26: 1616–23. 16452685 10.1523/JNEUROSCI.4964-05.2006 PMC6675489

[75] Maren S, Holt WG (2004) Hippocampus and Pavlovian fear conditioning in rats: muscimol infusions into the ventral, but not dorsal, hippocampus impair the acquisition of conditional freezing to an auditory conditional stimulus. Behav Neurosci 118: 97–110. 14979786 10.1037/0735-7044.118.1.97

[76] Bannerman DM, Grubb M, Deacon RM, Yee BK, Feldon J, et al (2003) Ventral hippocampal lesions affect anxiety but not spatial learning. Behav Brain Res 139: 197–213. 12642189 10.1016/s0166-4328(02)00268-1

[77] McNaughton N, Gray JA (2000) Anxiolyic action on the behavioural inhibition system implies multiple types of arousal contribute to anxiety. J Affect Disord 61: 161–76. 11163419 10.1016/s0165-0327(00)00344-x

[78] Bannerman DM, Rawlins JN, McHugh SB, Deacon RM, Yee BK, et al (2004) Regional dissociations within the hippocampus—memory and anxiety. Neurosci Biobehav Rev 28: 273–83. 15225971 10.1016/j.neubiorev.2004.03.004

[79] Kjelstrup KG, Tuvnes FA, Steffenach HA, Murison R, Moser EI, et al (2002) Reduced fear expression after lesions of the ventral hippocampus. Proc Natl Acad Sci USA 99: 10825–30. 12149439 10.1073/pnas.152112399 PMC125057

[80] Kopp SJ, Banisadr G, Glajch K, Maurer UE, Grünewald K, et al (2009) Infection of neurons and encephalitis after intracranial inoculation of herpes simplex virus requires the entry receptor nectin-1. Proc Natl Acad Sci U S A 106: 17916–20. 19805039 10.1073/pnas.0908892106 PMC2764878

[81] Haarr L, Shukla D, Rødahl E, Dal Canto MC, Spear PG (2001) Transcription from the gene encoding the herpesvirus entry receptor nectin-1 (HveC) in nervous tissue of adult mouse. Virology 287: 301–9. 11531408 10.1006/viro.2001.1041

[82] Horváth S, Prandovszky E, Kis Z, Krummenacher C, Eisenberg RJ, et al (2006) Spatiotemporal changes of the herpes simplex virus entry receptor nectin-1 in murine brain during postnatal development. J Neurovirol 12: 161–70. 16877297 10.1080/13550280600760594

[83] Baringer JR (2008) Herpes simplex infections of the nervous system. Neurol Clin 26: 657–74. 18657720 10.1016/j.ncl.2008.03.005

[84] Beers DR, Henkel JS, Kesner RP, Stroop WG (1995) Spatial recognition memory deficits without notable CNS pathology in rats following herpes simplex encephalitis. J Neurol Sci 131: 119–27. 7595636 10.1016/0022-510x(95)00099-n

[85] Gordon B, Selnes OA, Hart J Jr, Hanley DF, Whitley RJ (1990) Long-term cognitive sequelae of acyclovir-treated herpes simplex encephalitis. Arch Neurol 47: 646–7. 2346392 10.1001/archneur.1990.00530060054017

[86] Dickerson FB, Boronow JJ, Stallings C, Origoni AE, Cole S, et al (2004) Infection with herpes simplex virus type 1 is associated with cognitive deficits in bipolar disorder. Biol Psychiatry 55: 588–93. 15013827 10.1016/j.biopsych.2003.10.008

[87] Stiles KM, Milne RS, Cohen GH, Eisenberg RJ, Krummenacher C (2008) The herpes simplex virus receptor nectin-1 is down-regulated after trans-interaction with glycoprotein D. Virology 373: 98–111. 18076965 10.1016/j.virol.2007.11.012 PMC2629994

[88] Krummenacher C, Baribaud I, Eisenberg RJ, Cohen GH (2003) Cellular localization of nectin-1 and glycoprotein D during herpes simplex virus infection. J Virol 77: 8985–99. 12885915 10.1128/JVI.77.16.8985-8999.2003 PMC167240

